# Deep Margin Elevation – A Retrospective Clinical Study

**DOI:** 10.3290/j.jad.b5199089

**Published:** 2024-04-11

**Authors:** Ahmad M. El-Ma’aita, Heba Radwan, Mohammad A. Al-Rabab’ah

**Affiliations:** a Associate Professor, School of Dentistry, University of Jordan, Amman, Jordan. Research idea, performed the treatments, wrote the manuscript.; b Vocational Trainee, Jordan University Hospital, University of Jordan, Amman, Jordan. Data collection and processing.; c Associate Professor, School of Dentistry, University of Jordan, Amman, Jordan. Patient assessment, proofread the manuscript.

**Keywords:** subgingival margins, deep margin elevation, cervical margin relocation, restorative dentistry

## Abstract

**Purpose:**

The aim of this retrospective study was to assess the short- to mid-term restorative and periodontal outcome of deep margin elevation (DME) performed using resin composite.

**Materials and Methods:**

Twenty-eight teeth treated with DME and indirect adhesive restorations were followed-up for a mean of 25.4 months (minimum: 12 months). Clinical and radiographic examination assessed the adaptation of the DME material and indirect restorations, presence of recurrent caries or discoloration, periodontal health at DME and non-DME sites, and periapical health.

**Results:**

The overall success rate was 96.6%. One tooth showed signs and symptoms of apical pathology after 34 months following DME. No caries, discoloration, or periodontal pockets were detected in any of the treated teeth. DME had no detrimental effect on the gingival/periodontal health or plaque accumulation. There was no correlation between the distance from the DME material to the marginal bone level and pocket depth, gingival inflammation, and plaque accumulation (p > 0.05).

**Conclusion:**

Deep margin elevation might be considered a safe procedure for teeth with deep subgingival proximal caries in the short- and mid-term.

Extensive proximal cavities with subgingival margins are very common in clinical practice and dentists are often challenged with this restorative predicament.^18,34^ Deep subgingival margins are difficult to isolate, are often contaminated with saliva, blood and gingival crevicular fluid (GCF),^31^ and are difficult to capture with either conventional or digital impression techniques. Adhesive cementation of indirect restorations on teeth with subgingival margins is similarly difficult, and controlling excess cement is challenging.^26^

Different treatment options have been proposed to overcome the problems associated with deep subgingival margins. Surgical crown lengthening aims to expose the subgingival margins using either gingivectomy or an apically positioned flap, with or without crestal bone reduction.^16^ While considered predictable, it is an aggressive procedure that involves additional intervention, extra cost, and further delay of the definitive treatment. It can also be limited by the proximity of adjacent teeth and the presence/level of root furcations. Furthermore, it may compromise the crown:root ratio and can have a negative esthetic outcome.^16^ Orthodontic forced eruption is another method to expose the subgingival margins that is time consuming, costly, and requires strict patient compliance.^1^ Surgical extrusion (intra-alveolar transplantation) involves the intentional luxation of the root within its socket and its relocation at a more coronal position to expose the deep subgingival margin and provide a ferrule.^30^ However, it carries the risk of progressive root resorption and often necessitates root canal treatment in teeth with mature apices.^5^ Its success is also related to atraumatic luxation, which limits its application on multirooted teeth with diverging or curved roots.

Deep marginal elevation (DME) is a conservative treatment option that aims to relocate the cervical subgingival margin to a supragingival location using a direct restoration.^27,31^ As stated by Samartzi et al,^31^ “The rationale behind DME rests upon the coronal relocation of the restorative margin instead of displacing the margin of the periodontium according to the cavity limits”. It was first described by Dietschi and Spreafico in 1998^7^ and has since been referred to using different terms such as “proximal box elevation” and “cervical margin relocation”. DME facilitates rubber-dam isolation, moisture/bleeding control, impression taking, adhesive cementation of the indirect restoration, and predictable excess-cement control.^9^

However, evidence of the efficacy of DME is not unequivocal. In-vitro studies have yielded the bulk of data on DME performance, while well-designed and controlled clinical trials with long-term follow-up are lacking. Laboratory studies demonstrated that, compared with non-DME sites, DME had no detrimental effect on the quality of restorative margins, fracture resistance of the restored teeth, and bond strength to indirect restorations.^17,23,28^ Clinical data on DME are mainly limited to retrospective studies. Bresser et al^4^ reported a survival rate of 96% of 197 indirect restorations with DME after a 12-year follow-up period. Dietschi et al^8^ reported a 100% survival rate of 10 DME restorations after a mean follow-up period of 14 years. Indirect restorations with DME demonstrated a higher survival rate compared with surgical crown lengthening.^27^ While more bleeding-on-probing was detected at DME sites after one year,^11^ well-polished and finished DME restorations were shown to be compatible with periodontal health.^2,13,29^

Different materials were suggested for elevation of the subgingival margins. Glass-ionomer cement, resin-modified glass ionomer, and self-adhesive resin cement performed satisfactorily in laboratory studies.^12,15^ Resin composite (conventional, flowable and bulk-fill) was more extensively investigated as a DME material in-vitro and in-vivo.^9,21,31,35^ However, there is no consensus as to which material or technique is the most suitable for DME. The aim of this retrospective study was to assess the short- to mid-term restorative and periodontal outcome of DME performed using resin composite.

## Materials and Methods

**Fig 1:**
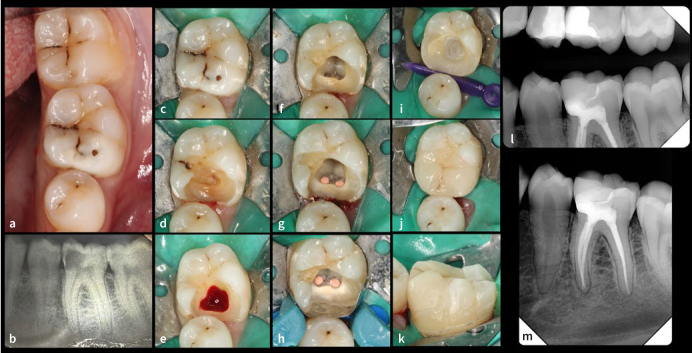
a) Clinical photograph and b) periapical radiograph of a mandibular left first molar with a deep mesio-occlucal cavity and signs and symptoms of irreversible pulpitis. c) Rubber-dam isolation using the cuff technique; d) initial caries excavation; e) access cavity following complete caries excavation showing inflamed pulp tissue; f) instrumented canals g) after obturation and showing the deep subgingival mesial margin; h) properly adapted sectional matrix band showing fluid-tight isolation; i) mesial margin elevated with resin composite; and j) and k) lithium-disilicate partial onlay cemented. Bitewing (l) and periapical (m) radiographs after the follow-up period showing good adaptation of the direct and indirect restorations, no recurrent caries and periodontal or periapical disease.

The study protocol was approved by the review board of Jordan University Hospital (ref.10/2021/17836). Patients who received a DME procedure between January 2019 and December 2021 were invited back for clinical and radiographic assessment after a follow-up period of at least 12 months. Informed consent was obtained from all participants. All the treated teeth had either primary or recurrent caries with one deep subgingival margin (mesial or distal, located >1 mm subgingivally) that otherwise would have required surgical crown lengthening, orthodontic or surgical extrusion, or extraction. The advantages, disadvantages, and alternatives to the DME procedure were explained to the patients before treatment. All the DME procedures and the indirect restorations were provided by the same operator (AE). Caries excavation was done with conventional diamond burs under rubber-dam isolation. For most cases, the cuff rubber-dam isolation technique was used and sectional metal matrix bands (Palodent Plus, Dentsply Sirona; Konstanz, Germany) were properly adapted to the cavity margins (Fig 1). However, in cases where the deep subgingival margins had no adjacent teeth (e.g., distal cavities of the most distal teeth), conventional rubber-dam isolation and circumferential matrix bands (Tofflemire matrix band system, PD Dental; Vevey, Switzerland) were used. Selective enamel etching was achieved with 37.5% phosphoric acid (Gel etchant, Kerr; Orange, CA, USA) for 20 s followed by thorough rinsing with a water jet and air drying. A layer of universal adhesive (Scotch bond Universal, 3M Oral Care; St Paul, MN, USA) was applied to both enamel and dentin, gently air thinned and light cured for 20 s using a Bluephase G2 polywave curing unit (Ivoclar Vivadent; Schann, Liechtenstein) at the soft-power setting (650–1200 mW/ cm^2^). Three or four layers of resin composite (Filtek Z250, 3M Oral Care) preheated at 55⁰C ± 1°C using a composite heating device (ENA-heat heater, Micerium; Uscio, Italy) were packed incrementally and light cured according to the manufacturer’s instructions, until the margin was elevated to a supragingival position (Fig 1). All the teeth were prepared for either partial or full-coverage indirect restorations as indicated by the clinical situation (i.e., quantity, quality and distribution of the remaining tooth structure, and the patient’s occlusion). Immediate dentin sealing was performed using a self-etching, 2-step adhesive (Clearfil SE Bond, Kuraray Noritake; Tokyo, Japan). Final light curing under glycerine gel was performed to minimize the presence of an oxygen-inhibited layer. Conventional impressions were taken with addition-reaction putty and wash silicone (Elite HD+, Zhermack; Badia Polesine, Italy), and temporization was done with an autopolymerizing temporary resin (Acrytemp, Zhermack) after isolating the prepared and sealed tooth surface with a layer of petroleum jelly. The indirect restorations were milled from either lithium-disilicate or monolithic zirconia ingots, as was deemed appropriate to the clinical situation (Table 1).

**Table 1:** Details of teeth restored with DME

Tooth (n)	Pulp vitality (n)	Indirect restoration	Indirect restoration material
Maxillary first premolar (2)	Vital (4)	Conventional crown (5)	LS2 porcelain (25)
Maxillary second premolar (1)	Non-Vital (24)	Endocrown (20)	Monolithic zirconia (3)
Mandibular second premolar (1)		Partial onlay (3)	
Maxillary first molar (9)			
Maxillary second molar (3)			
Mandibular first molar (9)			
Mandibular second molar (3)			
Total (28)	28	28	28

LS2: lithium disilicate.

Adhesive cementation of the indirect restoration was performed one week later, under conventional single or multiple-tooth rubber-dam isolation. After the fit try-in, the indirect restorations were conditioned according to a standard protocol.^10^ The lithium-disilicate restorations were acid etched with 5% hydrofluoric acid (Condac Porcelana, FGM; Joinville, SC, Brazil) for 20 s, rinsed with a water jet for 20 s, air dried, then coated with a silane coupling agent (silane primer, Kerr), and left to air dry for 1 min. The zirconia restorations were sandblasted using 50-μm alumina particles at 2 bar, coated with a zirconia primer (Z-Prime Plus, Bisco; Schaumburg, IL, USA), and dried with an air syringe for 5 s. The prepared and sealed tooth surfaces were cleaned using air-borne particle abrasion (APA) with 27-µm alumina (BioArt intraoral sandblaster; Barcelona, Spain), followed by enamel etching with 37% phosphoric acid for 20 s. A layer of a universal bonding adhesive (Scotchbond Universal, 3M Oral Care) was applied on the conditioned tooth surface, followed by a dual-cured resin cement (RelyX Ultimate, 3M Oral Care). The resin cement was initially light cured for 2 s on each side, excess cement was removed buccally, lingually, and interproximally, followed by light curing for 30 s on the buccal, lingual, and occlusal surfaces. The patients were then given routine oral hygiene instructions and were seen regularly for prophylaxis, but did not follow any specific oral hygiene program.

**Fig 2:**
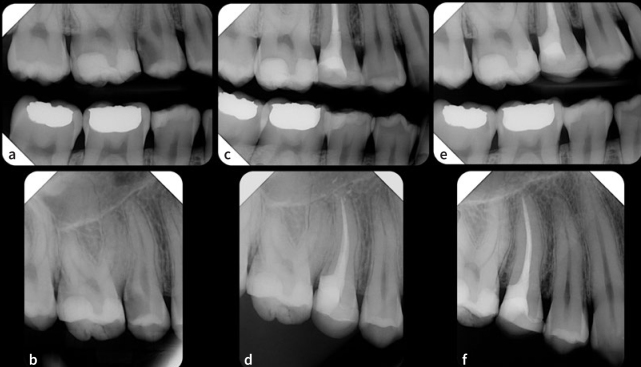
Preoperative bitewing (a) and periapical (b) radiographs showing the maxillary right second premolar with a deep distal cavity. A diagnosis of irreversible pulpitis with normal apical tissues was made. Following endodontic treatment, the distal margin was elevated with resin composite and the tooth restored with a lithium-disilicate overlay (c and d). After a follow-up period of 24 months, a bitewing (e) and periapical (f) radiographs show good adaptation of the DME material and the direct restoration, no recurrent caries or marginal bone loss, and the absence of apical pathology.

After the follow-up period, clinical examination was carried out by 2 experienced clinicians (MA and AE), and included assessing the adaptation of the DME resin composite and the indirect restoration to the tooth structure, the presence of recurrent caries, and discoloration of the tooth-restoration margins. Gingival/periodontal health was assessed around the DME and non-DME sites using a UNC periodontal probe (Hu-Friedy; Chicago, IL, USA) and included the assessment of plaque accumulation (Silness and Loe plaque index^22^), gingival inflammation (Silness and Loe gingival index), and pocket probing depth to the nearest 0.5 mm. Radiographic examination was carried out using periapical and bitewing radiographs (Fig 2) to assess the adaptation of both the marginal elevation material and indirect restoration, the presence of secondary caries or apical pathology, and the distance between the alveolar bone crest and the marginal elevation material. The correlation between the distance from the DME resin composite to the marginal bone level and the pocket depth, gingival inflammation, and plaque accumulation was tested using Pearson’s correlation coefficient.

Failure was defined as the emergence or persistence of signs and symptoms of apical or periodontal disease, or any outcome that required further intervention, including the development of recurrent caries, radiographic evidence of new apical pathology, and/or the need for surgical periodontal treatment. An asymptomatic tooth with an apical radiolucency that did not change in dimension or was reduced in size but not completely healed, and without any other adverse outcome, was considered a surviving tooth.

## Results

Twenty-five patients (14 females, 11 males) with 28 teeth treated with DME presented for assessment. Their age ranged from 19 to 66 years (mean: 40.4), and the follow-up time ranged from 12 to 47 months (mean 25.4). Twenty-four teeth were endodontically treated and 4 had vital pulps. Five teeth were restored with conventional crowns, 20 with endocrowns/overlays, and 3 with partial onlays. The details of the treated teeth are summarized in Table 1.

Clinical examination revealed good adaptation of the DME resin composite and indirect restorations in all the teeth assessed. None of the examined teeth had recurrent caries or marginal discoloration. Most DME sites were associated with healthy gingivae or mild gingival inflammation (Table 2). Only 4 DME and 4 non-DME sites showed moderate gingival inflammation with bleeding on probing (Silness-Loe grade 2). There was no statistically significant difference between gingival index scores around the DME and non-DME sites (Χ^2^ 2.1, p = 0.35). Pocket probing depth ranged from 2 to 3.5 mm, and there was no difference in the mean pocket depths between the DME and non-DME margins (mean: 2.8 and 2.6, respectively; t-test: 0.96. p = 0.34). Plaque accumulation was similar around the DME and non-DME sites (Table 2). The 4 teeth with a vital pulp presented no symptoms, responded normally to ethyl chloride cold testing, and showed no evidence of apical pathology.

**Table 2:** Summary of the findings of clinical and radiographic examination

Clinical examination	Adaptation			Good	Poor
DME material		28	0
Indirect restoration		28	0
Gingival index (Silness-Loe gingival index)		DME site	Non-DME site	Statistical test
	0	9	14	X^2^: 2.1 p = 0.35
	1	15	10	
	2	4	4	
	3	0	0	
Pocket probing depth (mm)		DME site	Non-DME site	Statistical test
	Range	2 - 3.5	2 - 3.5	t-test: 0.96 p = 0.34
	Mean	2.8	2.6	
Plaque accumulation (Silness-Loe plaque index)		DME site	Non-DME site	Statistical test
	0	6	7	X^2^: 0.10 p = 0.95
	1	19	18	
	2	3	3	
	3	0	0	
Radiographic examination	Adaptation			Good	Poor
		DME material		28	0
		Indirect restoration		28	0
	Recurrent caries			Absent	Present
		DME site		28	0
		Non-DME site		28	0
	Distance from DME margin to alveolar bone (mm)	Range		1 to 3	
		Mean		1.92	
	Radiographic evidence of apical pathology	Absent		27	
		Present		1	

X^2^: Chi-squared test.

**Fig 3:**
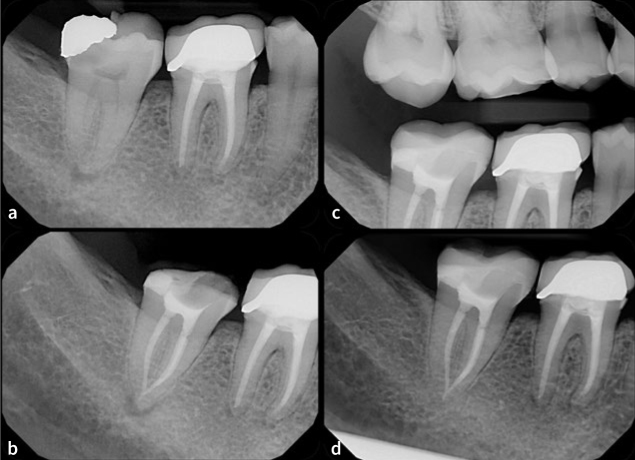
A periapical radiograph showing the mandibular right second molar with a defective disto-occlusal amalgam restoration, recurrent caries and a radiolucent apical lesion (a). A diagnosis of necrotic pulp with asymptomatic apical periodontitis was made. Root canal treatment and deep marginal elevation of the distal margin were performed (b). Following a follow-up period of 23 months, a bitewing (c) and periapical (d) radiographs show good adaptation of the DME material and the direct restoration, and the resolution of the apical pathology.

Upon radiographic examination, all teeth showed good adaptation of the DME resin composite and indirect restorations, and none showed evidence of recurrent caries development. The distance from the DME resin composite to the crest of the bone, measured on the bitewing radiographs to the nearest 0.5 mm, ranged from 1 to 3 mm (mean 1.92). Apical pathology was absent in 27 teeth (96.4%). Four teeth with pre-operative radiographic evidence of apical pathology showed complete resolution of the apical radiolucent lesion (Fig 3). One tooth was symptomatic upon presentation (acute apical abscess) and showed a new radiolucent lesion. Therefore, the overall success rate of DME in this study was 96.4% after a mean follow-up period of 25.4 months.

There was no correlation between the distance from the DME resin composite to the marginal bone level and pocket depth, gingival inflammation, and plaque accumulation (Pearson’s correlation coefficient: 0.15 [p= 0.44], 0.08 [p= 0.69], and -0.3 [p= 0.12], respectively).

## Discussion

Clinical data on DME are limited. Retrospective studies demonstrated high survival rates for DME^4,8^ and good compatiblity with periodontal health, provided the connective tissue component of the biological width was not violated.^2,11,13^ Samartzi et al^31^ suggested three criteria for a predictable DME procedure: complete isolation of the operating field, a well-adapted matrix band that perfectly fits around the margins and achieves a fluid-tight seal, and no violation of the connective tissue component of the biological width. Our results showed an excellent short- to mid-term outcome for DME, with a success rate of more than 96%. The fact that 4 teeth with preoperative apical pathology showed good healing upon follow-up suggests the presence of a good coronal seal. Upon follow-up, one tooth was symptomatic with evidence of apical pathology and was considered a failure. While coronal microleakage could be implicated, the outcome of endodontic treatment is multifactorial, and it is difficult to ascertain the exact cause of failure. The emergence of apical pathology in this case cannot be directly attributed to the DME procedure itself, especially since no recurrent caries could be identified clinically or radiographically in that tooth. The case was considered a failure and the patient opted to have the tooth extracted.

Pulp testing and thermal stimulation sensitivity were only performed on 4 teeth, as all others were endodontically treated. While these 4 teeth responded normally to pulp testing at the follow-up appointment, the effect of DME on pulp sensitivity merits further investigation through clinical trials. No detrimental effect of DME (i.e., caries development, discoloration, worsening peri-apical or periodontal health) was detected in our study. Safrati et al^32^ proposed that indications for surgical crown lengthening should decrease in the future, given that DME – despite being a demanding procedure – seems to be well tolerated by the surrounding periodontium.

The cuff rubber-dam isolation technique was preferred whenever possible during caries excavation and deep-margin build-up. The authors believe that rubber-dam slippage over the deep subgingival margin is common when the conventional single or multiple-tooth isolation techniques are used. Using the conventional isolation technique, the rubber-dam covering the papilla adjacent to the deep margin can be torn by the cutting bur, which could require rubber-dam replacement. Furthermore, the rubber-dam covering the interdental papilla adjacent to the deep margin can become entangled between the matrix band and cavity margin, which may result in poor adaptation of the composite and future microleakage. In contrast, the cuff technique provides good isolation of the operating field, permits caries excavation without tearing the rubber-dam, and allows proper matrix band adaptation to the tooth structure. The authors thus recommended this isolation technique during caries excavation and deep-margin build-up, whenever the clinical situation permits. However, during cementation of the indirect restorations, conventional single or multiple-tooth rubber-dam isolation was used, as the margins of the prepared teeth were supragingivally positioned at that stage. It is worth mentioning that another widely used technique to prevent damage to the rubber-dam and/or adjacent teeth and avoid injury to periodontal tissues is the use of oscillatory ultrasonic (or sonic) single-sided diamond tips.

The literature on DME shows that circumferential matrix bands (especially those with reduced height) have been recommended for DME.^20,32^ In this study, sectional matrix bands were used whenever there was adequate tooth structure buccally and lingually and an adjacent tooth to stabilize the matrix band using a wedge and a separation ring. The matrix band could be shaped with hand instruments to achieve the desired contour of the composite. The authors think it is easier to apply a sectional than a circumferential matrix band under rubber-dam isolation. However, in cases where there was no adjacent tooth (such as a distal cavity in the most distal tooth), or there was inadequate buccal and/or lingual tooth structure, a circumferential matrix band was used.

It is plausible to assume that the deeper the subgingival margin, the more difficult it is to adapt the matrix band and achieve fluid-tight isolation for composite placement. It may also be assumed that plaque accumulation and gingival inflammation can be more prevalent with deeper margins. In this study, all treatments were performed after meticulous matrix band adaptation and achievement of fluid-tight isolation. There was no correlation between the cavity depth (measured by distance from alveolar bone) and pocket depth, gingival inflammation, plaque accumulation, or apical pathology. This suggests that when DME is performed to a good standard, it is well tolerated by the periodontal tissues and can provide a good coronal seal. However, the authors acknowledge that in extremely deep subgingival cavities where ideal isolation and matrix band adaptation are not possible, DME should not be attempted. A poorly adapted restoration and contamination of the cavity margin with blood, saliva, or GCF results in an unfavorable environment for the periodontal tissues and defective bonding of the DME composite, which results in recurrent caries formation and coronal microleakage.

Resin composite offers a good option to restore missing tooth structure, as it is esthetic, possesses good mechanical properties, and can bond to tooth structure. However, it is a technique-sensitive material. Complete moisture control is key for its clinical success, and contamination with blood or saliva can impair its adhesion to tooth structure.^6^ Polymerization shrinkage is an inherent property of resin composites and can result in gap formation and microleakage.^14^ Furthermore, composites can be sticky, which makes its adaptation to the cavity walls challenging. While flowable composite can adapt better to the cavity margins, it was shown to degrade after thermomechanical loading and was therefore not recommended for DME.^33^ More recently, flowable composites with high filler loads have been introduced, with mechanical properties similar to conventional condensable resin composite (e.g., G-ænial Universal Injectable, GC Dental; Tokyo, Japan). They have the potential to be used as DME materials and can facilitate the procedure. Preheating resin composite can improve its handling properties and adaptation, and may reduce interlayer gaps.^3,26^ Preheated composite was associated with significantly less microleakage when used as a DME material compared with flowable and bulk-fill composite.^35^ In our study, heated microhybrid composite was used in combination with immediate dentin sealing (IDS) to elevate the subgingival margins. IDS was shown to improve the bond strength of indirect restorations to dentin, resulting in less gap formation and decreased bacterial leakage.^24,25^ Selective enamel etching in combination with a universal adhesive were used to avoid the risk of over-etching the dentin substrate in subgingival areas.^19^

DME remains a controversial subject. Dentists must ensure that they achieve complete isolation of the operating field, and use well-adapted matrix bands that achieve a fluid-tight seal for composite placement. They also need to understand bonding to tooth structure and appreciate the technique sensitivity of resin composites.

This study has certain limitations: the small number of patients, the limited criteria for patient selection, the short follow-up period, and its retrospective nature. DME is still a relatively new restorative option and is not yet well accepted by patients and dentists, which can limit patient recruitment. A longer follow-up period would allow the observation of long-term complications or negative outcomes related to DME. However, our results contribute to the available literature on DME and demonstrated that when performed to a high standard, DME can provide the restorative dentist with a conservative and predictable option to restore carious lesions with deep subgingival margins. Well-designed and controlled clinical trials with large samples and long-term follow-up are needed to address these uncertainties.

## Conclusion

DME demonstrated good short- to mid-term outcomes as a restorative option for teeth with deep, subgingival proximal carious lesions. More robust clinical evidence is needed through randomized controlled clinical trials with longer-term follow-up to strengthen the results of the present study.

## Clinical relevance:

The results of this study support the use of DME as a promising restorative option to restore carious with deep subgingival margins without resorting to complex and aggressive treatment options that add to the cost and time of the proposed treatment.
